# Cleaner production of essential oils from Indian basil, lemongrass and coriander leaves using ultrasonic and ohmic heating pre-treatment systems

**DOI:** 10.1038/s41598-023-31090-0

**Published:** 2023-03-17

**Authors:** Rajeev Kumar, Sangeeta Chopra, Anil K. Choudhary, Indra Mani, Shruti Yadav, Sukanya Barua

**Affiliations:** 1 Division of Agricultural Engineering, ICAR-Indian Agricultural Research Institute, New Delhi, 110012 India; 2 Division of Agronomy, ICAR-Indian Agricultural Research Institute, New Delhi, 110012 India; 3 Division of Agricultural Extension, ICAR-Indian Agricultural Research Institute, New Delhi, 110012 India; 4 Present Address: Division of Crop Production, ICAR-Central Potato Research Institute, Shimla, 171001 India

**Keywords:** Biochemistry, Plant sciences, Energy science and technology, Engineering

## Abstract

Indian basil (*Ocimum basillicum*), lemongrass (*Cymbopogon flexuosus*) and coriander (*Coriandrum sativum*) leaves are a good source of aromatic oils; however, their extraction volume is low. Hence, two pre-treatment systems (ohmic-heating and ultrasonic) were devised for extraction of essential oils (EO) from the leaves of these three plant spp., which consequently enhanced the EO yield and saved the time and energy. First of all, an experimental set-up was developed for ohmic-heating pre-treatment which was subjected to the optimization of electric conductivity of lemongrass and coriander leaves at 26.25 V/cm and for Indian basil at 22.5 V/cm voltage gradient. An Experimental setup was also developed for ohmic heating-assisted hydro-distillation (OHD). Finally, conventional Clevenger hydro-distillation (CHD), OHD, ultrasonic-assisted conventional hydro-distillation (UACHD) and ultrasonic-assisted ohmic-heating hydro-distillation (UAOHD) methods were evaluated for their effectiveness in the extraction of the EOs. The OHD took 3.5 h time with 410 W power consumption compared to 5 h time and 500 W power consumption in CHD of sleeted leaves. Likewise, a saving of ~ 86% in time and 74% in energy consumption was observed for EO extraction through UAOHD over CHD. Quantity of EOs extracted from all three aromatic plant spp. leaves followed the trend of UAOHD > UACHD > OHD > CHD methods, respectively. Overall, ultrasonic pre-treatment coupled with ohmic-heating assisted hydro-distillation (UAOHD) proved as an innovative and effective clean EO extraction technology which took shorter extraction time and lesser energy consumption with better EO yield over the UACHD, OHD and CHD methods from the leaves of Indian basil, lemongrass and coriander.

## Introduction

Global essential oil production is ~ 0.12 million metric tonnes (Mt) where India holds 3rd place with ~ 17% share after China and Brazil^1^. Essential oil (EO) is a complex mixture of volatile compounds having the characteristic fragrance of a plant from which it is extracted^2^. Essential oils are isolated from the specialized cells/glands (subcuticular spaces of glandular cells, organelles) of the aromatic plants by employing the physical methods for extraction^3,4^. In general, steam-distillation, hydro-distillation, hydro-diffusion and cold-pressing are the most commonly used methods in the oil extraction industry^2,5,6^. Mass transfer of target phyto-compounds inside the plant cell through the cell wall determines the extraction methods to be employed^5^. Biological membranes separating the inner cell contents from the outside, act as the prime barrier to diffusion while the concentration gradient acts as the driving force for diffusion across cell membranes and the cell walls. Hence, overcoming the separation of the cell wall can enhance the extractability of intracellular compounds for better EO yield.

Pre-treatment of aromatic plant materials by the exogenous electrical or ultrasonic field may facilitate the electroporation of biological membranes for higher EO release and their further separation by steam/water methods. Thus, OHD and ultrasonic pre-treatment systems may prove to clean EO extraction technologies for enhanced EO extraction in various aromatic plants over the conventional extraction methods, where most of the organic solvents used in conventional EO extraction processes cause air pollution being volatile and toxic in nature. Ohmic-heating hydro-distillation is a thermal process of internally generating uniform heat in a short span of time by the flow of alternating electrical current (AC) through heating bodies like plant material which produce electrical resistance^7–9^. The heating rate depends upon the electrical strength and conductivity in the heating body. It is possible to alter the electric strength by adjusting the spacing of electrodes at both ends of the heating body or by tuning the applied alternative current voltage. Ohmic heating-assisted hydro distillation is an advanced technique which uses an ohmic heating process for EO extraction with less power consumption per unit of EO extracted^6,10^. Similarly, ultrasonic-assisted extraction leads to higher EO extraction with lesser thermal degradation which ultimately produces EOs of high quality and essence despite comparatively less energy-use^3,11–14^.

In ultrasonic pre-treatment systems, the ultrasonic cavitation generates bubble implosion in leaf fluids to create shear forces in cell fluids, fluid streaming and micro-turbulence which collectively destroy essential oil glands of the plant cells/tissues and enhance the EO extraction^13^. Major benefits of the ultrasonic pre-treatment system lie in its precise control over ultrasonic intensity, temperature, pressure and retention time of pre-treatment etc. which enhance the quantity and quality of extracted EOs^3,11,15^. In general, the EO yield is ~ 1–1.5% in the raw plant material of aromatic crops like Indian basil (*Ocimum basillicum*), lemongrass (*Cymbopogon flexuosus*) and coriander (*Coriandrum sativum*). Hence, conventional EO extraction would prove more expensive, energy- and time-consuming if we follow the conventional distillation operating at low pressure and temperature recommendations. The EO extraction industry needs alternative safe and clean technologies to bypass the extraction procedures like distillation or solvent extraction or 3–4 times re-extraction from same lot of raw material so as to reduce the extraction time, energy and extraction costs. In contrast, the cell wall membrane rupturing with some pre-treatments would be advantageous to extract more quantity of EOs in lesser time using less energy and expenses. In addition, solvent-free extraction processes are now in great demand as most of the volatile organic solvents used in conventional EO extraction processes cause air pollution and toxicity^2^. Hence, the essential oil industry across the globe has shown great concern for the research and development on such advanced clean EO extraction technologies^1,16^.

Ohmic heating and ultrasonic pre-treatment systems may cause electroporation of cell membranes with improved oil extraction kinetics resulting in higher EO extraction in a shorter span of time. Dao et al.^17–19^ have also discussed the process kinetics on the extraction of EOs from lemon peel, pomelo peels and lemongrass leaves in details; which may greatly help in devising clean EO extraction technologies. Since, India is the largest producer of essential oils from Indian basil (*Ocimum basillicum*) and lemongrass (*Cymbopogon flexuosus*) with the production of about 500 and 150 t/annum, respectively; while essential oil production from coriander (*Coriandrum sativum*) is ~ 5 t/annum^20^. Hence, Indian basil, lemongrass and coriander were chosen as the test aromatic crops in the current study. Lemongrass EO has numerous bioactive compounds, such as citral, isoneral, isogeranial, geraniol, geranyl acetate, citronellal, citronellol, germacrene-D, and elemol. It has antifungal, antibacterial, antiviral, anticancer, and antioxidant properties^21^. The major constituents of lemongrass EO are neral (31.5%), citral (26.1%), and geranyl acetate (2.27%)^22^. Basil EO is extensively used in food products, perfumery, and dental and oral products. It also exhibits antimicrobial activity against a wide range of Gram-negative and Gram-positive bacteria, yeast, and molds^23^. Coriander EO shows highest collagenase, elastase, tyrosinase, and hyaluronidase inhibitory activities with very high level of linalool (81.29%) as the most abundant constituent^24^. Some research work has already been done on ohmic-heating pre-treatment systems for oil extraction from rice bran^25^, which is being used by the oil industry in India and overseas. Maize and oat flour ohmic heating is also in practice in few industrial pockets in India^26,27^. Review of literature has shown that there is no reported work on application of ohmic heating and ultrasonic field assisted extraction of essential oils from the leaves of these three plant spp. viz. Indian basil (*Ocimum basillicum*), lemongrass (*Cymbopogon flexuosus*) and coriander (*Coriandrum sativum*) in India and abroad. Hence, current study was planned to develop the ohmic-heating and ultrasound assisted pre-treatment systems which may lead to the development of an energy-efficient clean EO extraction technology from the leaves of Indian basil, lemongrass and coriander as well as other aromatic crops.

## Results and discussion

### Physical properties of selected plant spp. leaves

The physical parameters/properties of the leaves of Indian basil (*Ocimum basillicum*), lemongrass (*Cymbopogon flexuosus*) and coriander (*Coriandrum sativum*) harvested at optimal stage (peak vegetative stage), are presented in Table 1. In general, the length, width, thickness, leaf weight, leaf moisture content as well as bulk and true density of harvested leaves of these aromatic crops showed a wide variation amongst them (Table 1), which may be attributed to the genetic expression of these plant types^2,28^.

**Table 1:** Measurement of physical parameters/properties of leaves of selected plant spp.

Properties	Lemongrass	Basil	Coriander
Length (mm)	1036.52 ± 59.21	56.55 ± 4.01	34.45 ± 4.6
Width (mm)	19.96 ± 1.72	29.35 ± 3.01	29.55 ± 5.1
Thickness (mm)	0.24 ± 0.026	0.17 ± 0.07	0.24 ± 0.04
Weight/leaf (g)	1.77 ± 0.15	0.19 ± 0.032	0.19 ± 0.24
Bulk density (kg/m^3^)	100.25 ± 7.67	48.63 ± 4.88	89 ± 3.66
True density (kg/m^3^)	956.77 ± 6.89	1047.3 ± 5.66	889 ± 3.6
Moisture content (%)	70.28 ± 1.97	94.67 ± 3.79	97 ± 3.69

### Optimization of voltage gradient for effective ohmic-heating pre-treatment

**Figure 1:**
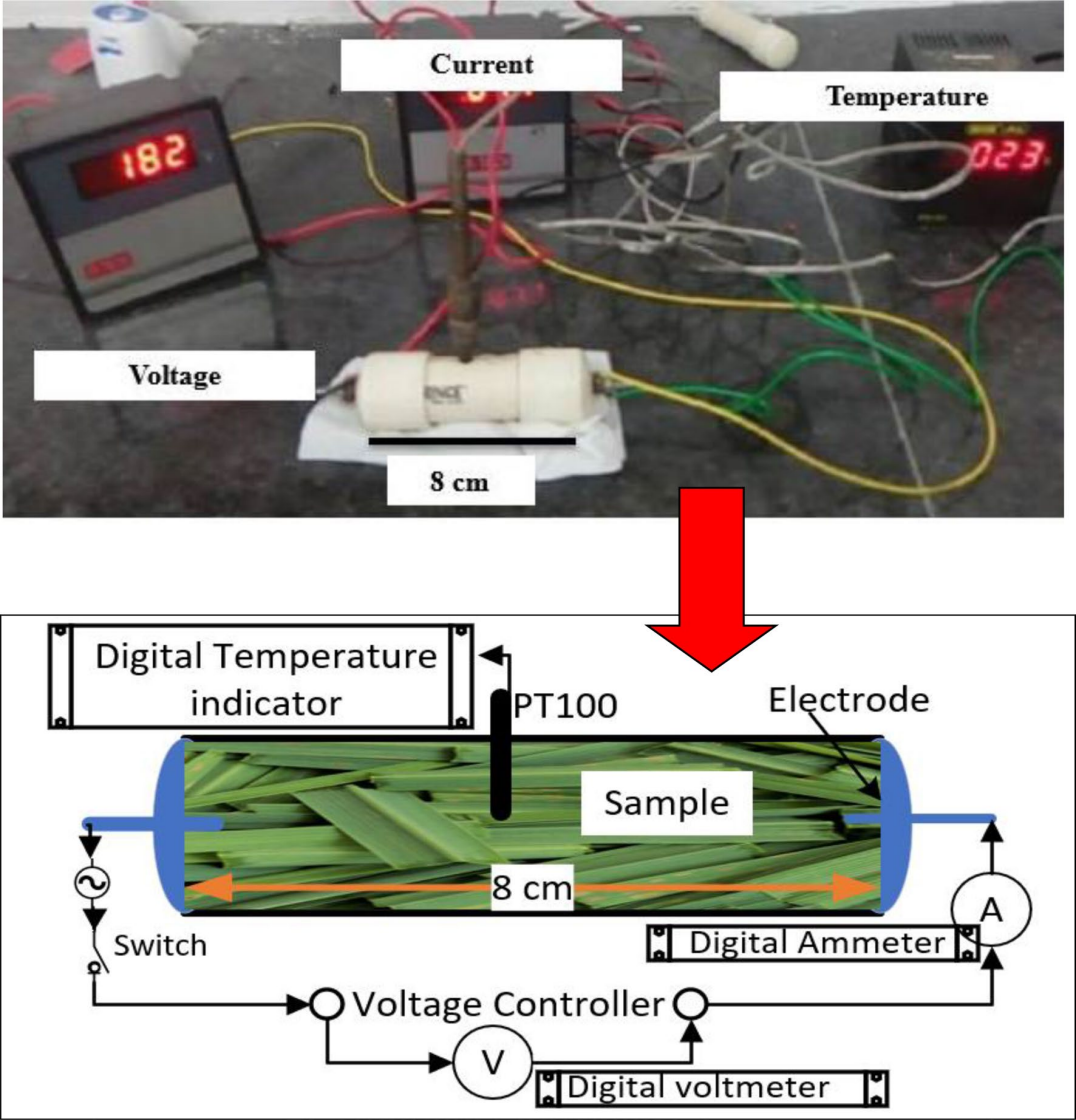
Experimental set-up for measurement of electrical conductivity.

**Figure 2:**
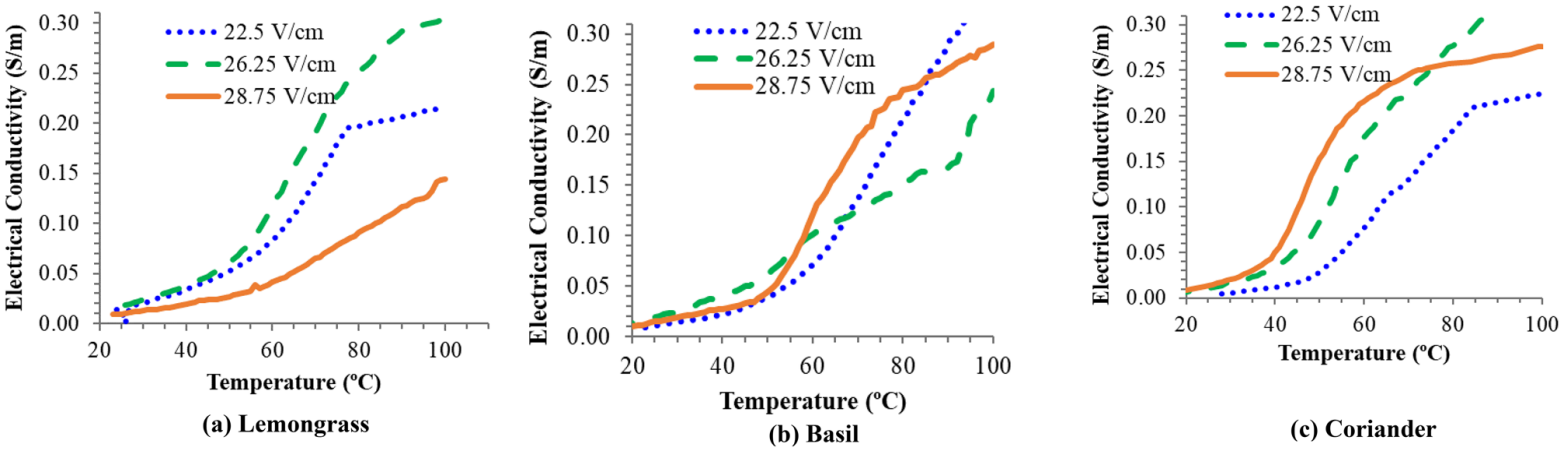
Graphical presentation of electrical conductivity × temperature relationship for: (**a**) Lemongrass, (**b**) basil, and (**c**) coriander leaves.

In the experimentation (Fig. 1), we optimized the voltage gradients for effective ohmic-heating pre-treatment of the leaves of all three aromatic plants. It was found that the electrical conductivity of the lemongrass (*Cymbopogon flexuosus*) leaves (test crop) increased with an increase in temperature at all three different voltage gradients viz*.* 22.5, 26.25 and 28.75 V/cm (Fig. 2).

**Figure 3:**
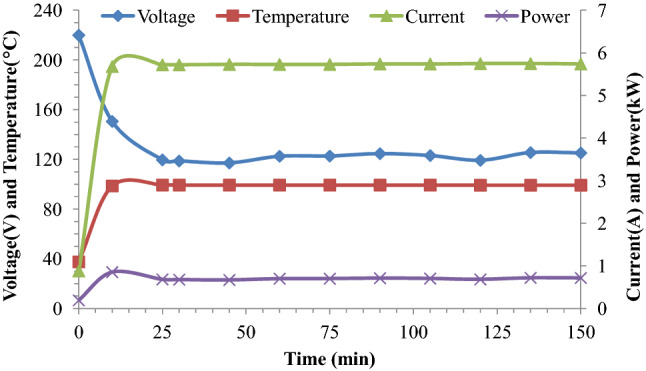
Variation in average electrical parameters and temperature during ohmic heating of lemongrass leaves.

The voltage gradients were calculated as the voltage applied across an 8 cm pipe length. The data in Fig. 1 shows that the electrical conductivity of lemongrass leaves proved optimal at a 26.25 V/cm voltage gradient for effective ohmic heating. Similarly, the electrical conductivity of Indian basil (*Ocimum basillicum*) and coriander (*Coriandrum sativum*) leaves showed a variation in temperature elevation at the 3 voltage gradients viz*.* 22.5, 26.25 and 28.75 V/cm (Fig. 2). The electrical conductivity of Indian basil and coriander leaves proved most suitable at 22.5 and 26.25 V/cm Voltage gradients, respectively for effective ohmic heating. Electrical conductivity is the major parameter which determines the heating rate of an ohmic process which obviously occurs due to more electrical energy input from the power supply and consequently conversion of electrical energy into heat energy due to the joule effect^16^. Variation in average electric parameters and temperature during ohmic-heating of lemongrass leaves as shown in Fig. 3, revealed that all these parameters became constant after 15 min of ohmic pre-treatment time. Similar trends were also found for Indian basil and coriander leaves for ohmic heating in the current study. Tunc and Koca^10^ have followed the similar procedure for the optimization of electrical conductivity for effective extraction of EOs from clove buds using an ohmic-heating pre-treatment system.

### Comparative performance of conventional clevenger vs ohmic heating hydro-distillation

**Figure 4:**
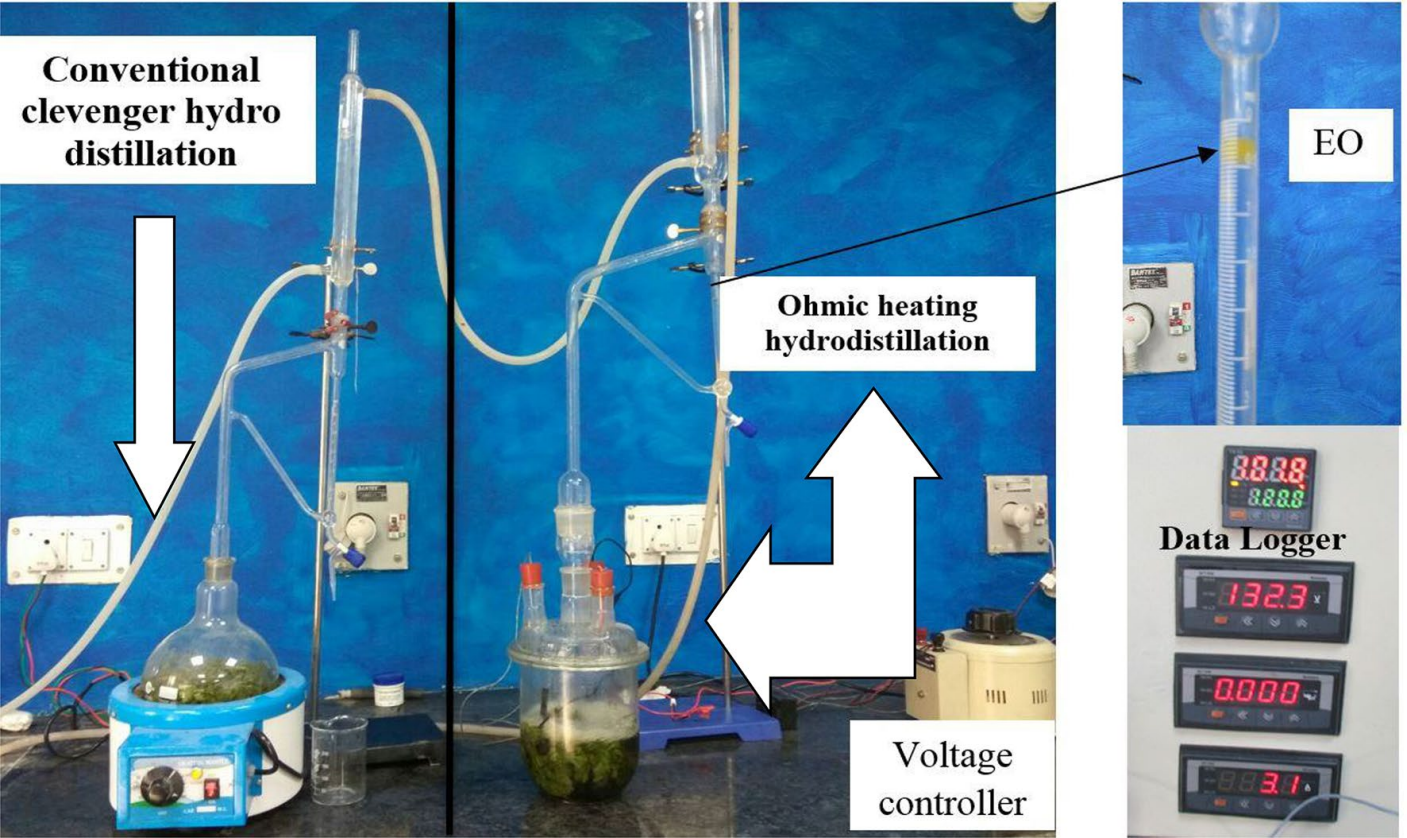
Conventional clevenger and ohmic heating hydro-distillation systems.

For studying the comparative performance of conventional clevenger and ohmic-heating hydro-distillation systems, the electric current, voltage and total time taken in EO extraction process and amount of EO extracted were measured in thrice replicated experimentation (Fig. 4).

The average power consumed during EO extraction was calculated using voltage and ampere data (Table 2). It was found that ohmic-heating assisted hydrodistillation (OHD) used ~ 64% less power with ~ 50% time saving compared to conventional Clevenger hydrodistillation (CHD). In OHD, the heat is generated very rapidly in all layers of the leaf sample with adequate electrical conductivity and is transferred quickly to other leaf constituents which results in sudden eruption of the essential oil glands almost according to Fourier’s law with least time energy use^16^. In ohmic heating, electrical energy was transformed to heat energy within the conductor (leaf) by applying an alternating current across the plant material in the PVC pipe chamber (Fig. 1). As, the applied energy was almost entirely dissipated within the heated plant material without heating the intervening heat exchange walls^29^, hence, the ohmic-heating pre-treatment was more energy-efficient with ~ 100% energy transfer efficiency^30^. Al-Hilphy^31^ also observed that OHD used lesser time and power consumption for EO extraction as compared to the conventional methods from dried eucalyptus leaves.

**Table 2:** Comparison of conventional clevenger hydro distillation (CHD) and ohmic-heating hydro distillation (OHD) processes.

Parameters	Conventional clevenger hydro-distillation (CHD)	Ohmic heating hydro-distillation (OHD)
Sample (g)	100	100
Time taken (h)	5	2.5
Power (kW)	0.5	0.610
Energy used (kWh)	2.5	1.525

### Comparative performance of ultrasonic conventional hydro-distillation (UACHD) vs ultrasonic ohmic hydro-distillation (UAOHD)

An ultrasonic pre-treatment instrument was selected for pre-heating of selected plant spp. leaves, before extraction of EOs using both conventional and ohmic-heating hydrodistillation methods. Comparative performance of ultrasonic conventional clevenger hydro-distillation (UACHD) and ultrasonic ohmic hydro-distillation (UAOHD) processes revealed that UAOHD proved its superiority over UACHD with respect to quantity of extracted EOs, extraction time, power use and energy consumption for EO extraction from the 100 g leaf sample of each selected plant spp., taken in triplicate (Table 3).

**Table 3:** Comparison of ultrasonic conventional hydro-distillation (UACHD) and ultrasonic ohmic hydro-distillation (UAOHD) processes.

Parameters	Ultrasonic clevenger hydro-distillation (UACHD)	Ultrasonic ohmic hydro-distillation (UAOHD)
Sample (g)	100	100
Time taken (min)	90	30
Power (kW)	0.5 kW	0.972 kW
Energy used (kWh)	0.75 kWh	0.486 kWh
Lemongrass (ml EO)	1.63^a^ ± 0.12	2.37^a^ ± 0.14
Basil (ml EO)	0.83^b^ ± 0.29	1.40^b^ ± 0.17
Coriander (ml EO)	0.50^c^ ± 0.04	0.65^c^ ± 0.07

Different letters (a, b, c) for EOs among the aromatic plants spp. indicate significant differences at *p* ≤ 0.05.

**Figure 5:**
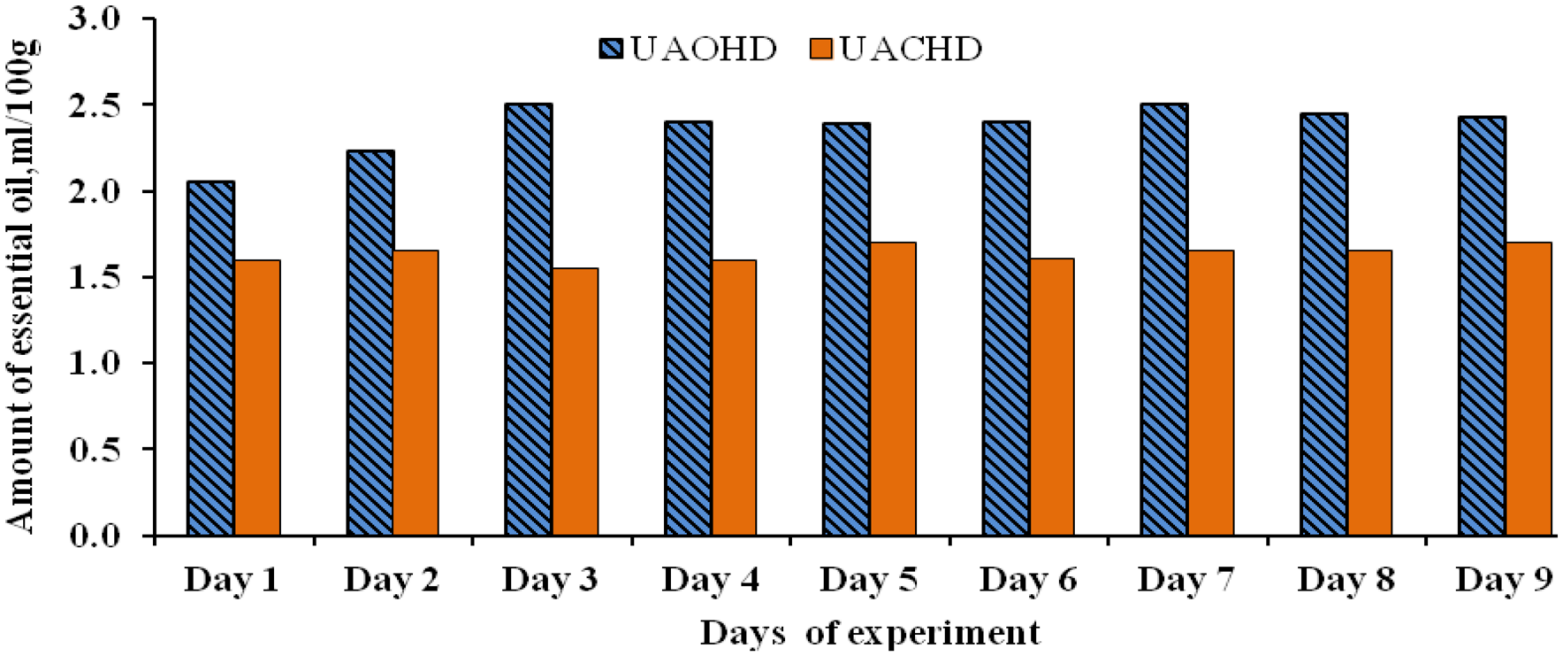
Amount of essential oil (EO) extracted by UAOHD and UACHD from lemongrass leaves in the different days of the experiment.

Quantity of EOs extracted in lemongrass using both ultrasonic pre-treatments assisted conventional Clevenger hydro-distillation (UACHD) and ohmic-heating hydro-distillation (UAOHD) as shown in Fig. 5; were subjected to one-way ANOVA analysis at 0.05 *α*-value at different days of EO extraction experimentation (Table 4). From Table 4, it is clear that there was no significant difference for extracted EOs among different days of experiments (*p* value > 0.05, and calculated *F-*value < *F-*critical). A similar trend was observed for the quantity of extracted EOs from basil and coriander leaves as well.

**Table 4:** ANOVA of the quantity of extracted EO of lemongrass through UAOHD and UACHD.

Source of variation	UAOHD					UACHD					
	SS	df	MS	F	*P*-value	SS	df	MS	F	*P*-value	F crit
Between groups	0.06	2	0.03	1.79	0.25	0.01	2	0.003	1.58	0.28	5.14
Within groups	0.11	6	0.02			0.01	6	0.002			
Total	0.17	8				0.02	8				

Where *SS* sum of square, *MS *mean sum of square, *df* degree of freedom.

In ohmic heating, the rapid generation of heat in leaf samples with adequate electrical conductivity leads to the sudden eruption of the essential oil glands resulting in higher EO extraction^10,16^. On the other hand, in ultrasonic pre-treatment systems, the ultrasonic waves create cavitation bubbles into the liquid medium of the plant material which results in its outbreaking due to huge energy generation^11^. This develops the shear forces in cell fluids and the plant material surface, thus, eroding its topmost layers and consequently leading to pore formation, fluid streaming and micro-turbulence due to the subsequent resonance of cavitation bubbles which collectively destroy the lipid glands of the plant cells/tissues and enhance the EO extraction^12^. This sonoporation further travels through the crack to the next layer and so on which overcomes the mass transfer barrier for easier and faster oil extraction with less energy consumption^32^, hence, making this process an environment-friendly and energy-efficient clean technology. In this way, ultrasound pre-treatment facilitates pouring out of jailed essential oils from the glands with less energy consumption over the conventional methods^4^. Therefore, the integration of both ultrasound and ohmic hydro-distillation under the UAOHD process proved highly effective over the UACHD with lesser time and energy input while giving higher EO extraction output in all the three aromatic plant spp. (Table 3).

### Influence of different pre-treatment processes/systems on essential oil extraction

**Figure 6:**
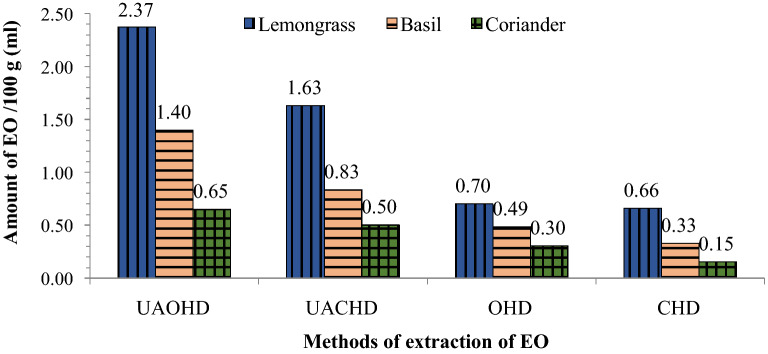
Influence of different pre-treatment technologies/processes on amount of essential oil (EO) extraction from lemongrass, Indian basil and coriander leaves.

The conventional Clevenger hydro-distillation (CHD), ohmic-heating assisted hydro-distillation (OHD), ultrasonic assisted conventional Clevenger hydro-distillation (UACHD) and ultrasonic assisted ohmic-heating hydro-distillation (UAOHD) methods were also evaluated for their effectiveness in the extraction of essential oils. It was found that UAOHD offers shorter isolation time and more EO extraction as compared to UACHD (Fig. 6). Da Porto and Decorti^11^ also observed good flavour and higher EO volume from spearmint plants using ultrasonic assisted hydro-distillation as compared to conventional methods. Since, pre-treatment of aromatic plant materials by the exogenous electrical or ultrasonic field lead to electroporation of biological membranes which results in higher EO release^11,12^. Here too, the selected plant leaves were pre-treated with an ultrasonic system and then EOs were extracted using ohmic-heating pre-treatment assisted hydrodistillation which led to the saving of ~ 86% time and ~ 74% energy consumption as compared to CHD as calculated from Tables 2 and 3 and ultrasonic pre-treatment time and energy values. Morsy^33^ and Chen et al.^34^ also advocated that the ultrasonic-assisted extraction process took a shorter time for higher EO yield than conventional methods. In our study, the quantity of extracted oil from lemongrass, Indian basil and coriander leaves increased in the order of CHD < OHD < UACHD < UAOHD, respectively (Fig. 6).

Thus, UAOHD showed significant potential for extraction of EOs from aromatic plant leaves which can be extended for EO extraction from seeds and herb roots/rhizomes as well. Positive effect of ultra-sonication on EO extraction from plant leaves may be attributed to the pre-treatment breakdown or rupturing of plant cells that resulted in faster and more extraction of EOs^11–14^. Overall, the superiority of UAOHD over the UACHD, OHD and CHD in our study indicates that the integration of ultrasound with ohmic hydro-distillation is an innovative extraction process which is highly effective in energy conservation with better EO yields which may have positive impact on the environment with lesser carbon footprints. Despite that, it may also possibly reduce the EO extraction time and costs making the process a green and sustainable EO extraction technology over the conventional methods.

## Conclusions

In current study, the electrical conductivity of both lemongrass and coriander leaves was optimized at 26.25 V/cm voltage gradients while for basil leaves it was optimal at 22.5 V/cm voltage gradient for effective ohmic-heating. Quantity of EOs extracted in all three aromatic plant spp. leaves followed the trend of UAOHD > UACHD > OHD > CHD methods, respectively; with the least energy consumption under the UAOHD process among the tested pre-treatment systems. UAOHD proved as an energy-efficient clean EO extraction technology which led to a saving of ~ 86% time and ~ 74% energy for EO extraction over the CHD. Overall, the UAOHD is an innovative process exhibiting significant potential for efficient EO extraction from the leaves of Indian basil, lemongrass and coriander. It is strongly recommended that UAOHD technology may extensively be used in the EO extraction for better EO yields from aromatic plant materials being faster and more energy-efficient.

## Materials and methods

### Planting of cultivars of selected leaves

The promising varieties of lemongrass (*Cymbopogon flexuosus*), basil (*Ocimum basillicum*), and coriander (*Coriandrum sativum*) viz. Pragathi, Anand Basil-1 and RCR-436, respectively were planted at the Research Farm of the Division of Agricultural Engineering, ICAR-Indian Agricultural Research Institute, New Delhi, India during *Rabi* season 2017 in the month of December following the recommended package of practices, under the supervision of an Agronomist. At the appropriate stage (peak vegetative stage), the leaves of these three aromatic plant spp. were harvested for current study i.e. lemongrass, basil and coriander were harvested after 120, 75 and 60 days after sowing, respectively. The length, width and thickness of harvested leaves were measured using digital Vernier-calliper least count of 0.01 mm^28^. Weight of leaves was then measured using digital weighing balance of 1 mg precision. Moisture content of leaves was measured using oven drying method. Bulk and true density of leaves were also measured using standard methods.

### Optimization of voltage gradient for effective ohmic-heating pre-treatment

An experimental set-up was developed for measurement of electrical conductivity which consisted of voltage, current and temperature measuring instrument. The conceptual layout of this set-up is presented in Fig. 1. Temperature inside PVC pipe was measured by the PT100 sensor. The current was measured using a current ammeter (CT, 30/5A) with signal ranging from 0 to 15 A. Temperature, electric current and voltage across the heated samples were displayed using a MT4W series indicator. Three different voltage (180, 210 and 230 V) were applied using voltage controller transformer (input: 240 V 50/60 Hz, Output: 0–270 V, 50/60 Hz, maximum load 10 Ampere) in the selected leaf samples kept inside the 8 cm long close PVC pipe having 20.4 mm internal diameter fitted with two tungsten electrodes at both ends of the pipe and subsequently the temperature was also recorded (Fig. 1). The voltage was applied across the PVC pipe ends till the temperature inside the pipe reached 100 °C. For the measurement of electrical conductivity, the leaves of all the three selected plant spp. were initially soaked in distilled water for 5 min before keeping the leaves inside the PVC pipe in triplicate samples each. Electrical conductivity of these leaf samples was then calculated using following equation^35^:

1 $$\sigma = \frac{L}{A} \times \frac{I}{V}$$

where σ, L, A, I and V are electrical conductivity (S/m), distance between electrodes (m) in the PVC pipe, cross-sectional area of PVC pipe (m^2^), alternating electric current passing through the sample (ampere), and voltage across the sample (Volts), respectively.

### Development of ohmic-heating pre-treatment system

An ohmic-heating pre-treatment system was developed for extraction of essential oils (EOs) as shown in Fig. 4. This system consisted of a four-neck flat bottom glass stream distillation flask of 2 L volume’, two tungsten electrodes inserted in two necks; while 3rd neck was used for receiving the extracted EO in graduated receiver tube of EO determination apparatus (Clevenger apparatus) for measuring the EO volume. The 4th neck of the above system was fitted with PT100 temperature sensor for monitoring the temperature changes in the ohmic-heating pre-treatment system (Fig. 4). Voltage controller was used to apply optimum voltage gradient corresponding to selected plant spp. leaves. A leaf sample of 100 g of each plant spp. in triplicate was pre-treated at optimum corresponding voltage gradients. Electric parameters such as voltage, electric current and temperature were recorded with the help of data logger (Fig. 4). Power consumption was also measured using voltage and ampere during the ohmic-heating of lemongrass, Indian basil and coriander leaves.

### Ultrasonic pre-treatment system

An ultrasonic pre-treatment instrument was selected for pre-heating of selected plant spp. leaves, before extraction of EOs using both conventional and ohmic-heating hydrodistillation methods. The specification of the ultrasonic pre-treatment instrument used is given in Table 5.

**Table 5:** Specification of digital ultrasonic pre-treatment system used.

Parameters	Specification
Tank volume (L)	4 L
Ultrasonic wattage	100 W
Ultrasonic frequency	40 ± 3 kHz
Tank dimension (taper)	235 mm × 135 mm × 150 mm
Heating	Ambient to 80 °C
Digital timer	5–60 min
Tank material	Stainless steel
Power supply	220 Volt, Ac 50 Hz, Single phase

The selected plant spp. leaves (100 g) were placed in the water-filled container bath of the ultrasonic pre-treatment instrument. It was then heated for a constant duration ~ 11 ± 1 min. Ultrasonic pre-heated leaves were then used for EO extraction using both conventional clevenger and ohmic-heating hydro-distillation methods. Comparative performance of ultrasonic conventional hydro-distillation (UACHD) and ultrasonic ohmic hydro-distillation (UAOHD) processes was also studied with respect to quantity of EOs extracted, extraction time, power-use and energy consumption for EO extraction from the 100 g leaf sample of each selected plant spp.

### Statistical analysis

All experiments were performed in triplicates. Analysis of variance (ANOVA) was done to determine significant differences between the means using SPSS statistical system (SPSS 20.0 for windows). Quantity of EOs extracted in lemongrass using both ultrasonic pre-treatments assisted conventional Clevenger hydro-distillation (UACHD) and ohmic-heating hydro-distillation (UAOHD) as a test crop were also subjected to one-way ANOVA analysis at 0.05 *α*-value at different days of EO extraction experimentation (*p* value > 0.05, and calculated* F* value < *F* critical).

### Research involving plants

It is stated that the current experimental research on the plants comply with the relevant institutional, national, and international guidelines and legislation. It is also stated that the appropriate permissions has been taken wherever necessary, for collection of plant specimens. It is also stated that the authors comply with the ‘IUCN Policy Statement on Research Involving Species at Risk of Extinction’ and the ‘Convention on the Trade in Endangered Species of Wild Fauna and Flora’.

## Data Availability

The datasets used and/or analysed during the current study available from the corresponding author on reasonable request.
